# Supplementary material for: Evaluation of Antioxidant and Anti-α-glucosidase Activities of Various Solvent Extracts and Major Bioactive Components from the Seeds of Myristica fragrans
Source: Molecules. 2020 Nov 8;25(21):5198. doi: 10.3390/molecules25215198 (PMC7664639; doi:10.3390/molecules25215198)
Supplement: Supplementary file 1 [file molecules-25-05198-s001.pdf]

Supplementary data

Evaluation of Antioxidant and Anti-α-glucosidase Activities of Various Solvent Extracts and Major Bioactive Components from the Seeds of *Myristica fragrans*

Cai-Wei Li [1,†], Yi-Cheng Chu [1,†], Chun-Yi Huang [2,†], Shu-Ling Fu [1] and Jih-Jung Chen [2,3,*]

[1] Institute of Traditional Medicine, National Yang-Ming University, Taipei 11221, Taiwan; leecw1219@gmail.com (C.-W.L.); xbox88888@gmail.com (Y.-C.C.); slfu@ym.edu.tw (S.-L.F.)
[2] Faculty of Pharmacy, School of Pharmaceutical Sciences, National Yang-Ming University, Taipei 11221, Taiwan; jimmyhuang9289@gmail.com (C.-Y.H.)
[3] Department of Medical Research, China Medical University Hospital, China Medical University, Taichung 40447, Taiwan
† Authors have contributed equally in this manuscript.
* Correspondence: chenjj@ym.edu.tw; Tel.:+886-2-2826-7195 (J.-J. Chen).

Contents

Table S1. Retention time, LOD, LOQ, and regression analysis for three components of *Myristica fragrans* in normal- and reversed-phase HPLC.......... S3

Figure S1. HPLC chromatogram of isolated compounds in normal- and reversed-phase chromatography.......... S3

Figure S2. Chromatogram of methanol extract in reversed-phase HPLC.......... S4

Figure S3. Chromatogram of ethanol extract in reversed-phase HPLC.......... S4

Figure S4. Chromatogram of acetone extract. in reversed-phase HPLC.......... S4

Figure S5. Chromatogram of ethyl acetate extract in reversed-phase HPLC.......... S4

Figure S6. Chromatogram of *n*-hexane extract in normal-phase HPLC.......... S5

Figure S7. Chromatogram of dichloromethane extract in normal-phase HPLC.......... S5

Figure S8. Chromatogram of chloroform extract in normal-phase HPLC.......... S5

Table S1. Retention time, LOD, LOQ, and regression analysis for three components of *Myristica fragrans* in normal- and reversed-phase HPLC.

Compounds	t_m (min)[a]	Regression equation, $Y = a + bX^b$		Correlation coefficient	LOD (μg/ml)[a]	LOQ (μg/ml)[a]
Noraml-phase		*a*	*b*			
Malabaricone B	33.45	5024.6	144020	0.9999	0.33	1.10
Malabaricone C	48.79	7529.9	103072	0.9999	0.49	1.62
Dehydrodiisoeugenol	23.57	238452	622458	0.9998	0.44	1.46
Reverse-phase		*a*	*b*			
Malabaricone B	37.75	17.8069	15.2535	0.9998	0.43	1.43
Malabaricone C	28.55	2.9053	9.56544	0.9996	0.38	1.25
Dehydrodiisoeugenol	27.34	16.7119	44.7684	0.9999	0.32	1.06

[a] t_m : Retention time ; LOD : Limit of detection ; LOQ : Limit of quantification
[b] Y refers to the peak area, X is concentration of the compounds (ug/ml)

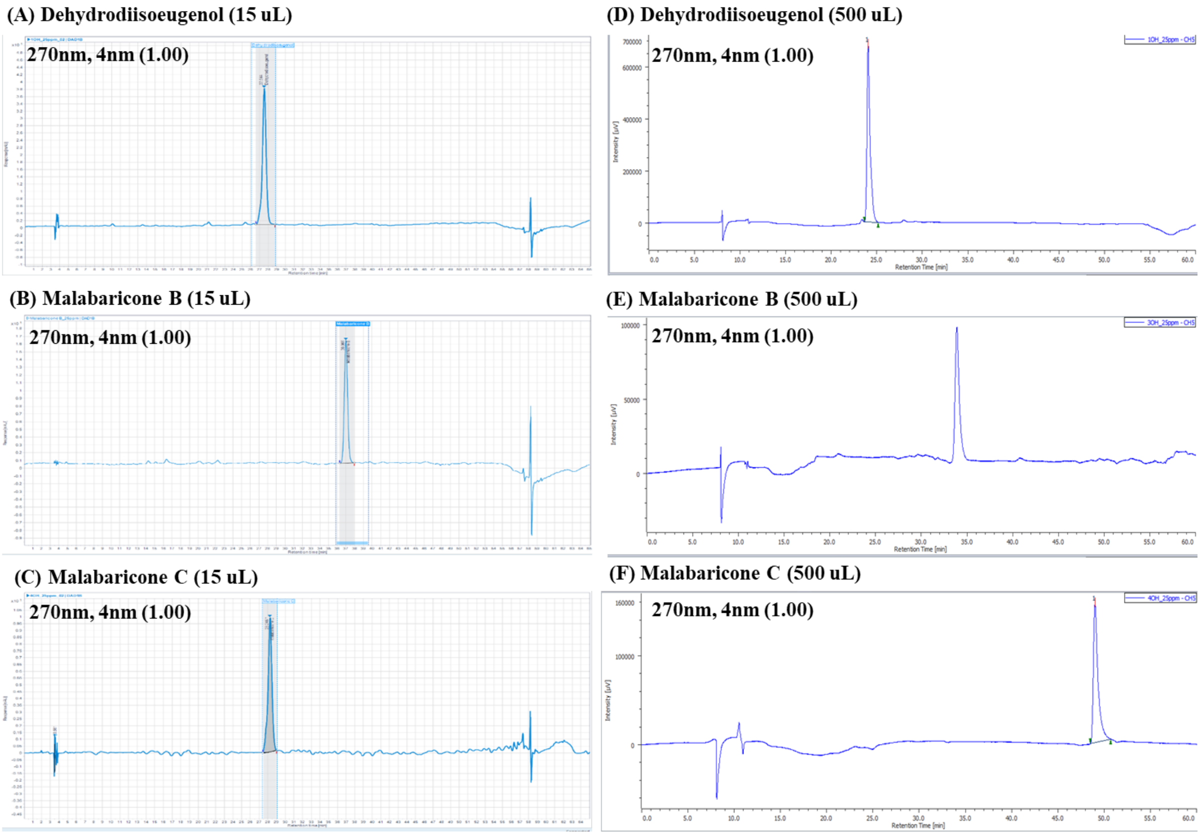

Figure S1. HPLC chromatogram of isolated compounds in reverse-phase (A to C) and normal-phase HPLC (D to F).

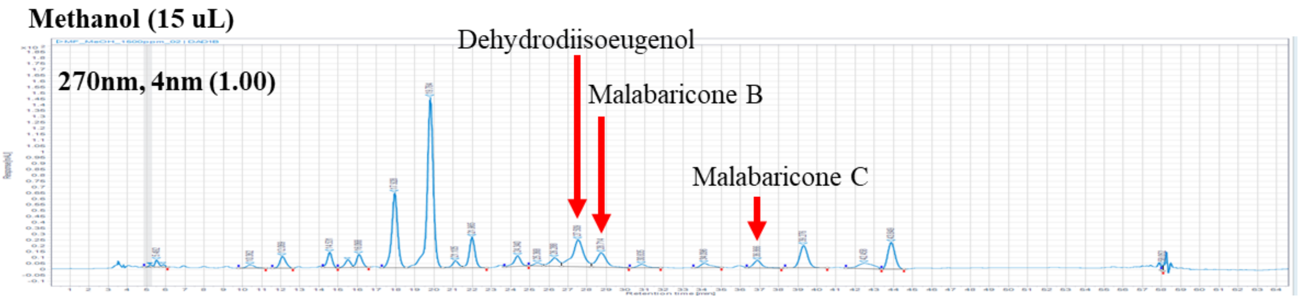

Figure S2. Chromatogram of methanol extract in reverse-phase HPLC.

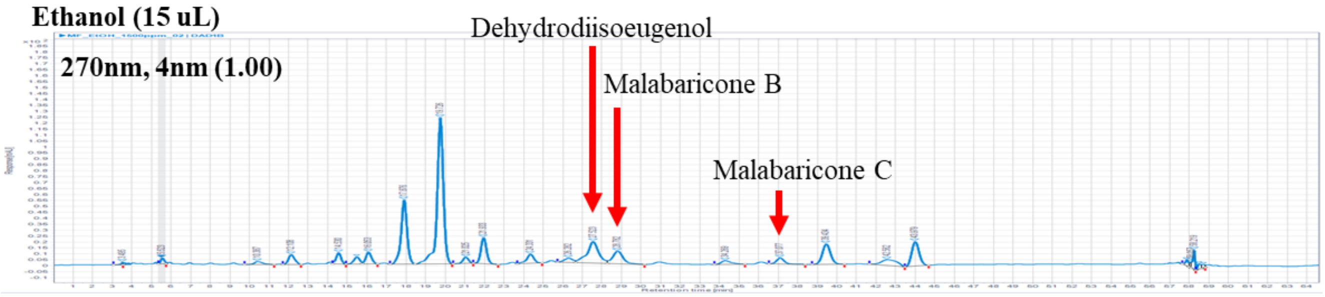

Figure S3. Chromatogram of ethanol extract in reverse-phase HPLC.

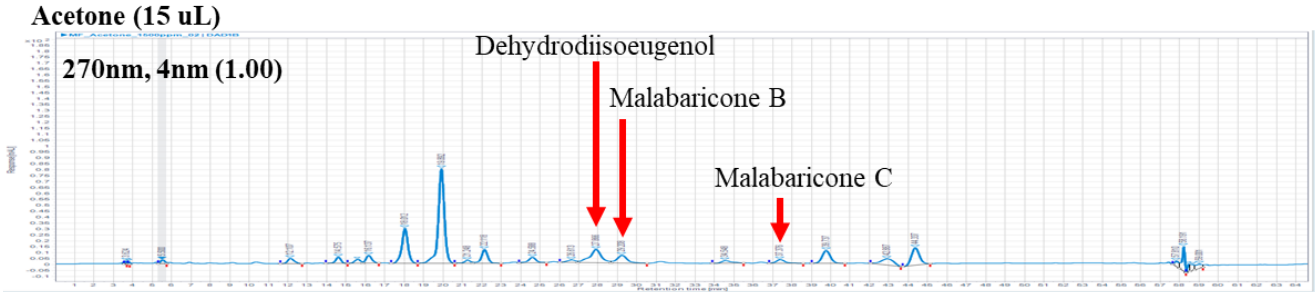

Figure S4. Chromatogram of acetone extract. in reverse-phase HPLC.

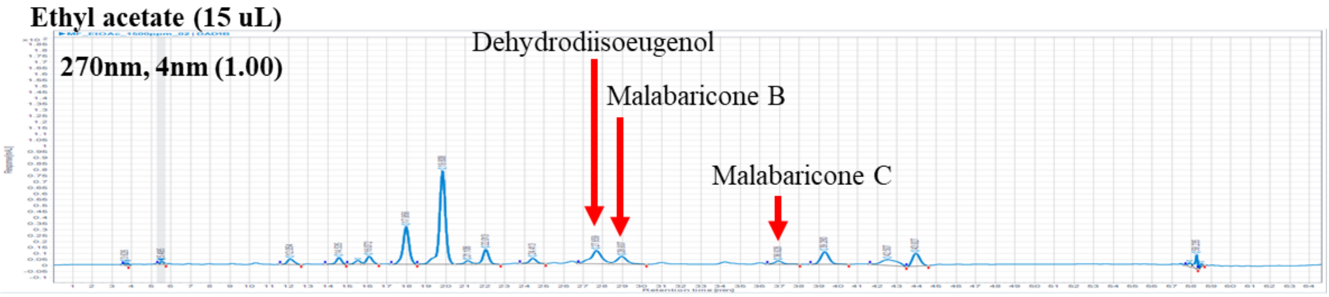

Figure S5. Chromatogram of ethyl acetate extract in reverse-phase HPLC.

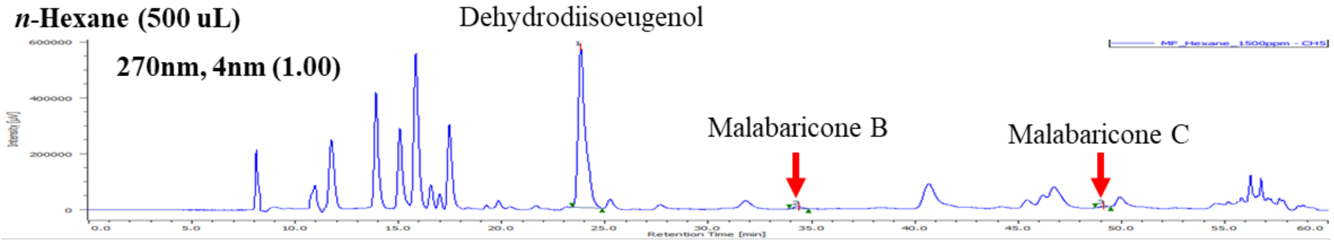

Figure S6. Chromatogram of *n*-hexane extract in normal-phase HPLC.

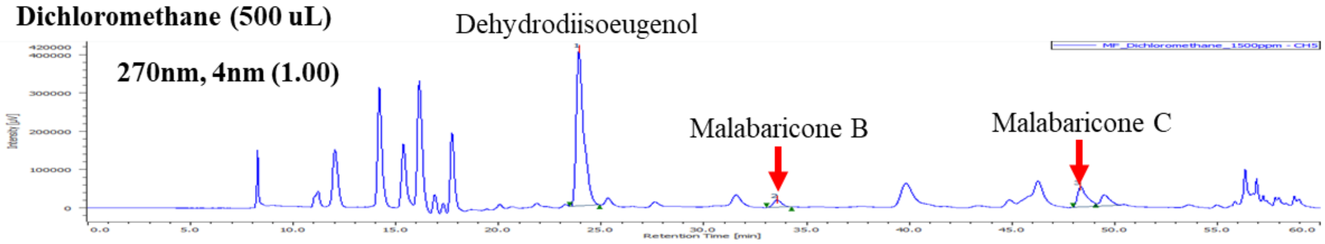

Figure S7. Chromatogram of dichloromethane extract in normal-phase HPLC.

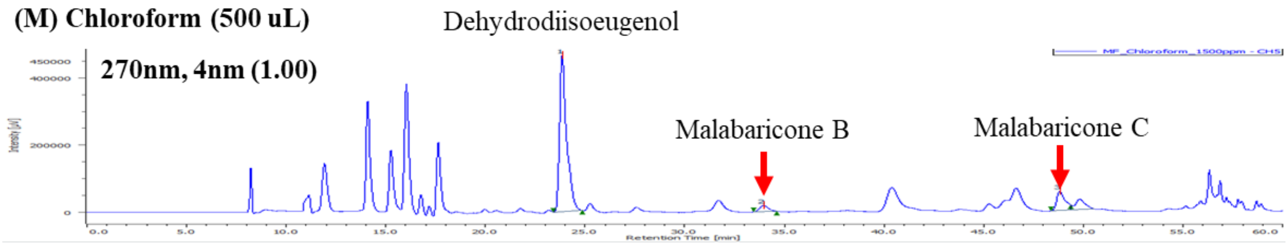

Figure S8. Chromatogram of chloroform extract in normal-phase HPLC.
